# Lifestyle interventions are feasible in patients with colorectal cancer with potential short-term health benefits: a systematic review

**DOI:** 10.1007/s00384-017-2797-5

**Published:** 2017-04-03

**Authors:** Susan J. Moug, Adam Bryce, Nanette Mutrie, Annie S. Anderson

**Affiliations:** 1 The University of Glasgow, Royal Alexandra Hospital, Corsebar Road, Paisley, PA2 9PN UK; 2 The University of Glasgow, University Avenue, Glasgow, G12 8QQ UK; 3 Physical Activity for Health Research Centre, Moray House School of Education, The University of Edinburgh, St. Leonard’s Land, Holyrood Road, Edinburgh, EH8 8AQ UK; 4 Centre for Public Health Nutrition Research, Division of Cancer Research, Ninewells Medical School, Level 7, Mailbox 7, Dundee, DD1 9SY UK

**Keywords:** Lifestyle interventions, Colorectal cancer, Patient outcomes

## Abstract

**Purpose:**

Lifestyle interventions have been proposed to improve cancer survivorship in patients with colorectal cancer (CRC), but with treatment pathways becoming increasingly multi-modal and prolonged, opportunities for interventions may be limited. This systematic review assessed the evidence for the feasibility of performing lifestyle interventions in CRC patients and evaluated any short- and long-term health benefits.

**Methods:**

Using PRISMA Guidelines, selected keywords identified randomised controlled studies (RCTs) of lifestyle interventions [smoking, alcohol, physical activity (PA) and diet/excess body weight] in CRC patients. These electronic databases were searched in June 2015: Dynamed, Cochrane Database, OVID MEDLINE, OVID EMBASE, and PEDro.

**Results:**

Fourteen RCTs were identified: PA RCTs (*n* = 10) consisted mainly of telephone-prompted walking or cycling interventions of varied durations, predominately in adjuvant setting; dietary/excess weight interventions RCTs (*n* = 4) focused on low-fat and/or high-fibre diets within a multi-modal lifestyle intervention. There were no reported RCTs in smoking or alcohol cessation/reduction. PA and/or dietary/excess weight interventions reported variable recruitment rates, but good adherence and retention/follow-up rates, leading to short-term improvements in dietary quality, physical, psychological and quality-of-life parameters. Only one study assessed long-term follow-up, finding significantly improved cancer-specific survival after dietary intervention.

**Conclusions:**

This is the first systematic review on lifestyle interventions in patients with CRC finding these interventions to be feasible with improvements in short-term health. Future work should focus on defining the optimal type of intervention (type, duration, timing and intensity) that not only leads to improved short-term outcomes but also assesses long-term survival.

## Introduction

The incidence of colorectal cancer (CRC) in the UK is increasing [1]. With neo-adjuvant and adjuvant multi-modal treatment pathways, complication risks and the possibility of a stoma, a patient’s physical and psychological recovery can be prolonged, resulting in reduced short- and long-term quality of life [2]. Additionally, recurrent disease will be diagnosed in ≥30% leading to poorer long-term survival [1, 2]. Therefore, there is a clear need to develop effective strategies that could improve the quality and duration of survivorship in CRC patients.

One such strategy is lifestyle interventions. The World Cancer Research Fund, The European Code against Cancer and The International Agency for Research into Cancer have presented a body of evidence demonstrating that almost a third of cancers can be prevented by improving the key lifestyle factors of excess weight, poor diet, smoking, alcohol excess and physical inactivity [1–6]. In addition, there is evolving observational and interventional evidence that modifying these factors can lead to improved peri-operative outcomes and, in the short-term, better quality of life for patients with cancer [7–14].

Lifestyle interventions can be methodologically complex, especially if more than one lifestyle factor is being assessed. As a consequence, the majority of work has been carried out in other cancer populations rather than CRC, which, with its multi-modal treatment options, can make the performing of randomised controlled trials (RCTs) difficult [11, 14]. To date, it is uncertain if RCTs on lifestyle interventions are feasible and/or beneficial in CRC populations.

This systematic review aims to collate the published evidence for the feasibility of performing lifestyle interventions in patients with CRC, allowing conclusions on the short- and long-term health benefits to be drawn.

## Materials and methods

The study was developed according to the PRISMA Guidelines [15], with guidance from the Cochrane Collaboration Handbook [16] using the PICO framework [17]. The protocol was registered on PROSPERO (CRD42015017205) [18].

### PICOS

Participants: adults ≥18 years of age with non-metastatic CRC.

Interventions: modification of ≥1 lifestyle factor (weight, diet, physical activity [PA], smoking and alcohol).

Primary outcomes: to assess feasibility of performing lifestyle interventions in CRC patients (e.g. recruitment, adherence and follow-up/retention rates)

Secondary outcomes: summarise any documented short (e.g. quality-of-life measurements) and long-term health outcomes (e.g. cancer recurrence, cancer-specific survival)

Study design: randomised controlled trials (RCTs)

### Search strategy

In June 2015, a clinical librarian searched the following databases to identify relevant publications: Dynamed, Cochrane Database of Systematic Reviews, OVID MEDLINE, OVID EMBASE, PEDro. Keywords used are the following: colon cancer, rectal cancer, colorectal cancer, obesity, diet, excess weight, smoking, alcohol, physical activity, exercise, randomised controlled trials, systematic reviews, lifestyle factors, lifestyle intervention, recurrence, disease-free survival, overall survival, short-term outcomes, long-term outcomes. Exclusion keywords were: risk factors, screening uptake, screening awareness. Clinicaltrials.gov website was searched for relevant registered trials. There were no restrictions on date or language of publication. Expert opinions, letters and comments were excluded.

### Screening and data extraction

The resultant citations (abstracts) were independently examined with duplicates removed (SJM and AB). After screening, the full text was obtained and those papers independently assessed for eligibility, with attention paid to reference lists of reviews. Each reviewer then independently allocated relevant papers to the four lifestyle factor sections: alcohol, diet/excess weight, PA and smoking. A paper could be allocated to more than one section if more than one lifestyle factor was assessed. For further information, the authors were contacted by e-mail and any reviewing disagreements were resolved by discussion or by conferring with one further reviewer (ASA or NM).

### Assessment of methodological quality

The Jadad scale [19] was applied to each paper to assess the methodological quality of the included trials. The quality scale involved three items: randomization, double blinding and withdrawals or dropout. The score ranged from 0 to 5: 0–2 for randomization, 0–2 for blinding and 0–1 for withdrawals or dropout, with a score of 2 or less considered methodologically poor [20]. Data were collected independently by the two reviewers and crosschecked.

## Results

**Fig. 1:**
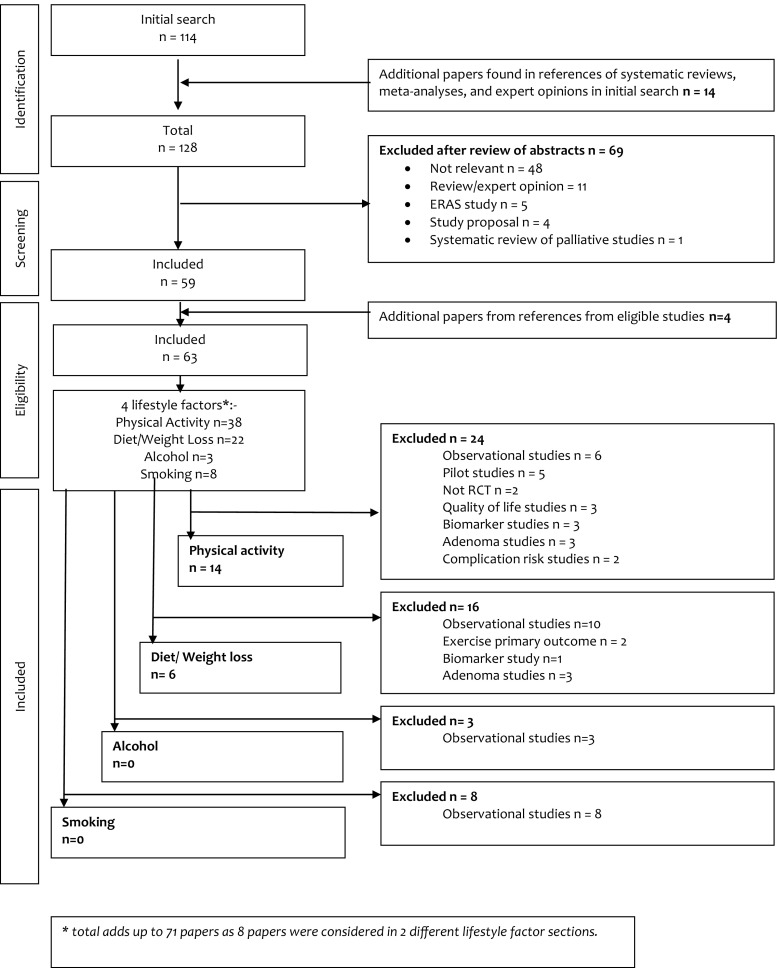
Flow diagram for the study selection process (PRISMA)

One hundred fourteen papers were identified after the literature search (Fig. 1). After searching the references of systematic reviews and meta-analyses, a further 14 papers were added. On review of the abstracts, 69 papers were excluded, leaving 59 full papers that were read with four additional papers found.

At the end of identification, screening and eligibility, there were no published RCTs on performing interventions in smoking and alcohol reduction/cessation, 10 RCTs in PA (14 published papers) and 4 RCTs in diet/excess weight (6 published works) [Tables 1, 2, 3 and 4].

**Table 1:** Description of RCTs on physical activity interventions in patients with colorectal cancer

Lead author	Country	Date published (start-end)	Journal	No. of centres	Age of population Mean (range or SD)	Jadad score
Adamsen (21)	Denmark	2009 (2004–2007)	BMJ	2	Intervention: 47.2 (21–65)Control: 47.2 (20–65)	3
Campbell (NC STRIDES) (22)	USA	2009 (2001–2004)	Annals of Behavioural Medicine	Multiple (Registry of 33 counties)	Defined according to ×4 groups^a^: Controls: 66.6 (SD 10.1) Tailored Printed Communication (TPC): 66.2 (10.5) Telephone Motivational Interviewing (TMI): 67.1 (SD 9.5) Combined: 65.9 (SD 9.8)	2
Carli (23) Mayo (24)	Canada	2010, 2011 (2005–2006)	BJS, Surgery	1	Intervention: 61 (SD 16) Control: 60 (SD 15)	1
Courneya (25)	Canada	2003 (1998–2001)	EJCC	1	Intervention: 59.92(SD 10.73) Control: 61.13 (SD 9.93)	3
Hawkes (CanChange) (26); Lynch (28)	Australia	2013; 2014 (2009 – n/a)	Journal of Clinical Oncology	Multiple (cancer registry)	Intervention: 64.9 (SD 10.8) Control: 67.8 (9.2)	3
Houborg (27,28)	Denmark	2005; 2006 (1999–2001)	EJCN; Scandinavian Journal of Surgery	3	Intervention: 72 (SD 6.5) Control: 72 (SD 7.3)	2
Kim (29)	Canada	2009 (n/s)	Tohoku Journal of Experimental medicine	1	Intervention: 55 (SD 15) Control: 65 (SD 9)	3
Ligibel (AACT) (30)	USA	2012 (2007–2009)	Breast Cancer Research and Treatment	10	Intervention: 53.1 (SD 10.8) Control: 55.5 (SD 10.6)	3
Morey (31) Demark-Wahnefried (RENEW) (32)	Canada, UK, USA	2009, 2012 (2005–2007)	JAMA; JCO	Multiple (cancer registries)	Intervention: 73.0 (SD 5.0) Control: 73.1 (SD 5.1)	3
Pinto (33)	USA	2011	Psycho-Oncology	1	Intervention: 59.5 (SD 11.2) Control: 55.6 (SD 8.24)	2

*SD* standard deviation

^a^Four groups contain both cancer survivors and healthy controls; no separate ages available

**Table 2:**
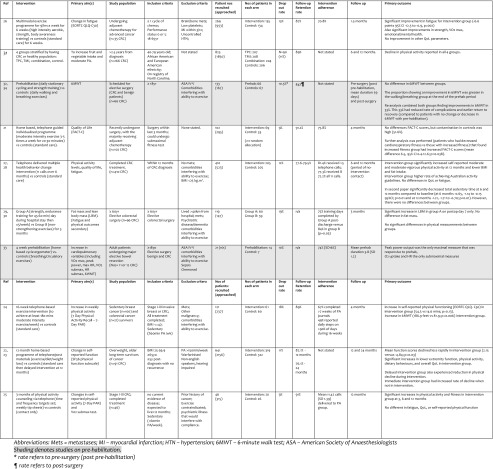
Aims and outcomes of RCTs on physical activity interventions in patients with colorectal cancer

Shading denotes studies on pre-habilitation

*Mets* metastases*, MI* myocardial infarction*, HTN* hypertension, *6MWT* 6-min walk test, *ASA* American Society of Anaesthesiologists

^a^Rate refers to pre-surgery (post pre-habilitation)

^b^Rate refers to post-surgery

**Table 3:** Description of RCTs on weight loss and diet interventions in patients with colorectal cancer

Lead author	Country	Date published (start-end)	Journal	No. of Centres	Age of population mean (range or SD^a^)	Jadad score
Campbell (NC STRIDES) (22)	USA	2009 (2001–2004)	Annals of Behavioural Medicine	Multiple (Registry of 33 counties)	Defined according to ×4 groups^b^: Controls: 66.6 (SD 10.1) Tailored Printed Communication (TPC): 66.2 (10.5) Telephone Motivational Interviewing (TMI): 67.1 (SD 9.5) Combined: 65.9 (SD 9.8)	2
Hawkes (CanChange) (26)	Australia	2013 (2009–n/a)	JCO	Multiple (cancer registries)	Intervention: 64.9 (SD 10.8) Control: 67.8 (9.2)	3
Morey (31); Demark-Wahnefried (RENEW) (23)	Canada, UK, USA	2009; 2012 (2005–2007)	JAMA; JCO	Multiple (cancer registries)	Controls 73.1 (SD 5.1) Intervention 73.0 (SD 5.0)	3
Ravasco (34,35)	Portugal	2005; 2012 (2000–2003)	JCO; American Journal of Clinical Nutrition	1	64.6 years (47–77)	3

^a^SD denotes standard deviation

^b^Four groups contain both cancer survivors and healthy controls; no separate ages available

**Table 4:**
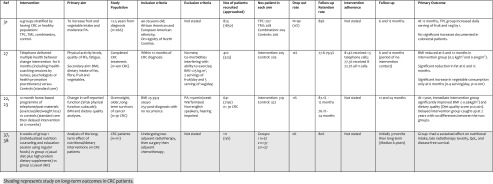
Aims and outcomes of RCTs on weight loss and diet interventions in patients with colorectal cancer

Shading represents study on long-term outcomes in CRC patients

## Physical activity and colorectal cancer

The 14 publications on physical activity (PA) intervention are mainly from North America, published from 2003 onwards, with the majority being: multi-centred in design, Jadad score 3, telephone-guided home/outpatient-based physical activities (cycling, walking) and performed as rehabilitation (adjuvant) rather than pre-habilitation (neo-adjuvant) (Tables 1 and 2). Recruitment rates vary, adherence is reasonable (range 67 to 81.4%) and follow-up/retention rates are reported as at least 80% in the majority of studies. Improvements in fatigue, physical activity parameters (e.g. 6-min walk test distance, submaximal fitness test and lower extremity function) and overall quality of life were documented in interventions ranging from 4 to 12 months. No study assessed cancer-free or overall survival [21–33, 36].

### Pre-habilitation in colorectal cancer patients

With the success of enhanced recovery after surgery programmes that focus on early mobility after surgery [37], researchers assessed pre-operative PA interventions (‘pre-habilitation’). The two published studies support the feasibility of performing a pre-operative physical activity intervention in patients with CRC; however, both studies contained mixed benign and malignant populations [23, 29]. In addition, colonic cancer accounted for the majority of CRC patients recruited. The exercise intervention was stationary cycling in both studies with mean duration of intervention completed 7.4 and 3.8 weeks respectively. Low dropout rates were reported (10.5 and 26%) with one study reporting an 84% follow-up rate, suggesting the interventions are tolerated well by patents. In relation to physical fitness improvements after the cycling intervention, one study reported no differences in 6-min walk test (6MWT) between groups at the two time points tested: pre-surgery (at the end of pre-habilitation) or post-surgery [mean 9.6 weeks, SD 3.4] [23]. In comparison, the other study found significantly improved cardiorespiratory function with an increased peak power (26% increase versus 0% in controls), reduced heart rate and oxygen uptake in their intervention group only [29]. It is possible that the study of Carli et al. was contaminated by their control group, increasing their 6MWT (pre-habilitation −10.6 m versus controls +8.7 m), a phenomenon described by Courneya et al. in 2003, where controls can be indirectly incentivised to exercise more [25]. Alternatively, the authors argued that the controls were not an optimal comparative group as their walking programme encouraged a minimum of 30 min/day, which means many will have achieved more than the weekly recommended UK guidelines for PA [38]. As a result, this group went on to re-analyse their data, combining the two groups to determine the overall affect of pre-habilitation on 6WMT, finding increases in 33%, no change in 38% and decreases in 29% [24]. These results translated in to significantly improved re-operative rates (29% in those that had deteriorated compared to the increased or no change groups combined, 18 and 2% respectively) and earlier recovery to baseline function 2–4 months after undergoing surgery (32 versus 77 versus 59%).

### Post-treatment PA interventions in colorectal cancer patients

This is the lifestyle area where most research has been performed. Courneya et al. (2003) published the first RCT assessing PA intervention in CRC survivors (74% colon cancer) that had completed their treatment at least 3 months previously [25]. For approximately 16 weeks, patients were randomised to a home-based exercise programme (cycling, swimming or walking) and weekly telephone calls whilst the controls received no exercise prescription but did receive weekly phone calls. Recruitment was only 35% with a high retention rate of 92%, suggesting the intervention was well tolerated. At follow-up testing, there were no differences in QoL measurements or PA between groups (FACT-C mean difference −1.3, 95% CI −7.8 to 5.1, *p* = 0.679). In addition to the study not being powered, the authors proposed that contamination by the control group could explain the results (documented at 51.6%) and explored this by performing an ancillary analysis comparing those patients that had increased physical fitness to those that had decreased. This time significantly improved QoL scores in the increased physical fitness group were found (6.5, 95%CI 0.4–12.6, *p* = 0.038).

The next four trials support the findings of Courneya et al. with a home-based telephone-guided PA intervention [26, 30–33]. The patient populations vary with some containing mixed cancer populations, and it is not always stated what percentage of the CRC patients recruited had rectal cancer. The exact interventions vary and can contain aerobic and strength components, making direct comparisons between studies difficult. However, low dropout rates (9–19%) with good adherence and retention rates suggest these interventions are feasible and well tolerated by CRC patients.

The majority of these studies have documented improvements in their physical parameters in CRC patients that had undergone adjuvant PA intervention. In the RENEW trial, older and overweight cancer survivors had significantly increased levels of activity, with the mean function scores and lower extremity function declining less rapidly in comparison to the control group that had delayed intervention after 12 months [31, 32]. At 2-year follow-up, the rate of decline in physical function had significantly slowed (seen in both the immediate and delayed intervention groups), but increased in the year after the intervention finished in the immediate group, displaying a protective effect of PA. Other studies have reported improved 6MWT (increased by 186.9 ft versus 81.9 in controls, *p* = 0.006), VO2 max (mean difference 0.16 L/min, 95% CI 0.1–0.2; *p* < 0.001) and leg press strength (29.7 kg, 23.4 to 34.9, *P* < 0.0001) [30, 33]. In addition, one study reported improved fatigue levels in the intervention group (EORTC QLQ C20; −6.6 points, 95% CI −12.3 to −0.9; *p* = 0.02). [30, 33]. At 1 year, the intervention group had.

The most recent publication on one of the largest powered trials on colon cancer participants found increased time in moderate PA at 12 months (30 min a day more; *p* = 0.003) [26]. In addition, the intervention group was more likely to achieve the Australian Physical Activity Recommendations (16.4 versus 9.2%; *p* = 0.047). Within the telephone counselling, advice was given about ‘limiting sedentary habits such as watching television’, and further work from this group found reduced sedentary time in both groups, but this difference was not significant [36]. However, a subgroup analysis found only the intervention group decreased sedentary time at 12 months in the >60 years of age, male and non-obese. The remaining three papers did not report any beneficial outcomes with adjuvant PA interventions in CRC patients which may reflect the different PA interventions. One group started in hospital training (walking on ward, stair climbing and strength training) that continued after discharge (×5/week) [27, 28]. There were no differences between groups at 30 and 90 days after surgery in sit-to-stand test and 6MWT. The authors suggested that post-operative exercise is not beneficial in the CRC population, but this study’s intervention was primarily based on strength exercises with the majority of the aerobic activity performed in the hospital to allow quick recovery of mobility post-operatively, rather than as part of a targeted progressive intervention.

The third paper that did not report improvements aimed to assess two different methods of promoting diet and PA: tailored print communication (TPC) only, telephone motivational interviewing (TMI) only, combination and controls [22]. Using self-reporting, the authors found that none of the four groups had an increase in PA at 1 year follow-up. This study was not powered, and the health behaviour interventions had substantially less patient contact compared to the other RCTs (e.g. TMI consisted of quarterly calls versus biweekly calls by CanChange) [26].

## Diet and excess weight in patients with colorectal cancer

These six publications represent four RCTs of reasonable methodological quality (mainly Jadad 3) that have been published in the last 10 years, originating from North America, Europe, Australia and Japan (Tables 3 and 4). Three were included in the PA section as they adopted a multiple lifestyle intervention approach, and the same three papers performed their dietary/weight intervention in the adjuvant setting. Study size varied from 91 to 825 with variable recruitment rates (30–78%). High retention rates were reported (at least 76%), but only one study quoted intervention adherence rates (>72%). All aimed to increase dietary quality in the short term, with one study assessing the influence of dietary change on disease-free survival [22, 26, 31, 32, 34, 35].

### Specific dietary interventions in colorectal cancer patients

Only one group has specifically assessed dietary interventions in rectal cancer, finding both short- and long-term patient benefits [34, 35]. Each patient underwent neo-adjuvant chemoradiotherapy for 1.5 months, surgery 3–5 weeks later, followed by adjuvant chemotherapy. During their radiotherapy, the authors randomised the patients into three groups: group 1 had 6 weeks of individualised nutrition counselling and education sessions using regular foods, group 2 had usual diet with high-protein supplements added in (40 g/protein/ day) or group 3 maintenance of usual diet. On completion of radiotherapy, groups 1 and 2 found significant increases in energy and protein intake, but at 3 months, only group 1 documented significant reductions in radiotherapy toxicity and improved nutritional intake/status and QoL. At 5-year follow-up [35], they found that again, only group 1 had a sustained improvement in dietary interventions with 91% maintaining adequate nutritional status. In addition to higher QoL scores, late radiotherapy toxicity was also significantly lower (9 versus 59% group 2 versus 65% group 3; *p* = 0.001). However, the most intriguing finding was that both disease-free survival and disease-specific survival were found to be significantly longer in group 1 after adjustment for age and disease stage (median survival: group 1 with 7.3 years versus group 2 with 6.5 years versus group 3 with 4.9 years; *p* < 0.01).

### Multiple behavioural interventions, including dietary interventions, in colorectal cancer patients

The next three RCTs performed dietary interventions in the adjuvant setting as part of a multiple behavioural intervention (e.g. physical activity, counselling), which is a pragmatic approach to lifestyle change [22, 26, 31, 32]. The time after diagnosis that the study started recruiting varied from the earliest (12 months) to the latest (8 years) with different patient populations in each study (one mixed cancer populations, one colon cancer patients only, one colon and rectal but numbers in each not stated). All interventions were based upon dietary counselling with home-based telephone guidance and reported low dropout rates (0–12%) with good follow-up rates (77.6 to 89%) supporting that these interventions were feasible and well tolerated.

Two of these three studies reported dietary improvement at follow-up [31, 32]. The RENEW Trial reported improved BMI and dietary quality in their older, overweight cancer patients (*n* = 91) [BMI −0.56 kg/m^2^ (95%CI −0.75 to −0.36; *p* < 0.001); Diet Quality Score 5.2 (95%CI 3.4 to 7.0; *p* < 0.001)]. These changes were maintained at the 2-year follow-up. In addition, their control group underwent similar improved dietary changes when they underwent their delayed intervention 12 months after the intervention group.

CanChange is the largest study in colon cancer patients finding good adherence to their telephone-guided counselling and [26] a significantly reduced BMI at 6 and 12-month follow-up as well as a reduction in fat intake (by 8.5% at 6 months and 7% at 12 months; *p* = 0.001 and *p* = 0.006) and increased vegetable intake (0.4 servings a day at 6 months; *p* = 0.001). Although this improvement intake seems small, it has the potential to be clinically relevant, as an increase of one portion of fruit and vegetables daily has been shown to reduce cancer.

NC Strides differs from the other two trials as it compared two types of behavioural intervention approaches to modifying lifestyle habits in patients with CRC [22]. TPC consisted of sending out four personalised newsletters. In comparison, TMI participants (telephone motivational interviewing intervention) received four telephone calls with specially trained motivational counsellors. After 1 year, there was a significant increase in fruit and vegetable consumption in the entire study population, but when the CRC patients were separately analysed (healthy controls excluded), there were no significant consumption improvements. One explanation could be selection bias as some participants had been recruited from another trial that had provided dietary advice at its completion.

## Discussion

Current multi-modal treatment pathways for patients with CRC are individualised, vary in duration and can include major resectional surgery, formation of a stoma, radiotherapy and/or chemotherapy. The challenge for initiating and maintaining lifestyle interventions in this patient group is clear, making it paramount that the feasibility of these trials is established. This systematic review has found that lifestyle interventions on physical activity and/or diet and weight are feasible in patients with colorectal cancer and can be performed peri-operatively, post-operatively and even many years after completion of cancer treatment, with the potential to achieve health benefits. This is the first systematic review to focus on RCTs in a colorectal cancer population, assessing all types of lifestyle interventions, not just one factor, and by including neo-adjuvant and adjuvant interventions [39–42].

Recruitment, intervention adherence and follow-up/retention rates were assessed to allow conclusions to be drawn on feasibility of lifestyle RCTs in CRC patients. Specific conclusions on the recruitment rates are difficult because several studies included other cancer populations, had small numbers of rectal cancer patients, had patients with benign colorectal disease or are, in some cases, not clearly reported. However, in one of the largest RCTs on 410 colon cancer patients, a recruitment rate of 78% for their multi-factorial lifestyle intervention performed in the adjuvant setting was reported [26]. Furthermore, of the 523 approached, only 5% declined suggesting that this intervention was attractive for colon cancer patients.

Adherence rates to the intervention were more commonly documented in the PA trials rather than the dietary/weight interventions (where only one RCT documented a rate of >72%) [26]. In PA interventions, reasonable adherence rates were reported from 67 to 81.4%. The majority of interventions were home-based telephone interventions rather than regular group-based sessions, which may be why adherence was good as patients could work their PA around their daily routine, including hospital appointments and treatments.

The duration of follow-up for all studies varied according to the study design being as short as 3.8 weeks (pre-habilitation study) to a minimum of 5 years. Within this range, follow-up rates vary from 77.6 to 91%, with the majority (9/14) reporting high follow-up rates of over 80%. It is worth highlighting that even the pre-habilitation studies, which had the tightest timeline to perform their PA intervention, had dropout rates of only 10%, supporting the feasibility of pre-habilitation [23, 30].

As patients with CRC make their way through their treatment pathway, many will experience side effects or significant changes to their health, including fatigue, reduced PA, altered emotional/mental health and reductions in their quality of life. Such changes can be acute, acute-on-chronic or chronic, and although they can exist independently, many cluster together resulting in significant impairment to a patient’s short- and long-term recovery [43]. This review has found that dietary/weight and/or PA interventions have the potential to significantly improve these symptoms, complementing findings that have been reported in other cancer populations [39, 44, 45]. How long these QoL improvements persist for is uncertain with only one dietary intervention assessing and reporting improved long-term outcomes [35].

Improvements in PA and strength measurements were reported in the majority of the 14 RCTs. The range of PA interventions on display, accompanied by a range of aerobic and strength tests, suggests that many types of PA interventions are feasible in CRC patients, but the type, duration and intensity of intervention that optimally improve health remain uncertain, a conclusion that other authors assessing other cancer populations have also drawn [46, 47]. As always, defining the ‘optimal intervention’ will need to take account of the patient as an individual and his/her’s planned oncological treatment pathway, and perhaps several options will need to be made open to allow each patient to select what is suitable for them. In addition, what denotes health will need to be defined, as it means many things to different cancer specialists. For example, surgeons will be interested in pre-habilitation as there is a possibility of ‘fitter’ patients having shortened length of hospital stay and reduced complications [23, 24]. Oncologists will be interested in reducing side effects and, therefore, improving quality of life as their patients progress through radio- and/or chemotherapy. Everyone, including patients, will be interested in long-term cancer survival, and to date, no RCT on PA intervention has assessed peri-operative or long-term outcomes, making these key areas of research focus. Certainly, it seems achievable to develop minimal recommendations of PA intervention specific to patients with colorectal cancer that are modified to the individual’s needs, and it is possible that the international multi-centred Colon Health and Life-Long Exercise Change Trial (CHALLENGE) may provide long-term answers for patients with colon cancer [48].

## Limitations of this review

Of the reviewed papers, none performed interventions on smoking and alcohol, making this an unknown area for patients with CRC. The other area of limitation is that many studies documented recruitment of patients with ‘colorectal’ cancer. However, the actual numbers of rectal cancer patients were poorly documented with the majority being colonic. Future work should define these different populations and the treatment received. Finally, due to the methodological heterogeneity, a meta-analysis could not be performed.

## Conclusion

This is the first systematic review that has shown that despite the demands of multi-modal treatment pathways for CRC patients, excess weight, dietary and PA interventions are feasible and acceptable in this patient population. With short-term psychological, physical, dietary and weight improvements reported, future trials should focus on optimising lifestyle interventions that integrate with CRC treatment pathways, allowing determination of their potential influence on long-term cancer-related outcomes.
